# Visual influences on ferret auditory cortex

**DOI:** 10.1016/j.heares.2009.06.017

**Published:** 2009-12

**Authors:** Jennifer K. Bizley, Andrew J. King

**Affiliations:** Department of Physiology, Anatomy and Genetics, University of Oxford, Parks Road, Oxford OX1 3PT, UK

**Keywords:** Spatial processing, Multisensory, Vision, Audition, Mutual information, Auditory cortex, Visual cortex, Auditory–visual, Anatomy, Spatial

## Abstract

Multisensory neurons are now known to be widespread in low-level regions of the cortex usually thought of as being responsible for modality-specific processing. The auditory cortex provides a particularly striking example of this, exhibiting responses to both visual and somatosensory stimulation. Single-neuron recording studies in ferrets have shown that each of auditory fields that have been characterized using physiological and anatomical criteria also receives visual inputs, with the incidence of visually-sensitive neurons ranging from 15% to 20% in the primary areas to around 50% or more in higher-level areas. Although some neurons exhibit spiking responses to visual stimulation, these inputs often have subthreshold influences that modulate the responses of the cortical neurons to sound. Insights into the possible role played by the visual inputs can be obtained by examining their sources of origin and the way in which they alter the processing capabilities of neurons in the auditory cortex. These studies suggest that one of the functions of the visual input to auditory cortex is to sharpen the relatively imprecise spatial coding typically found there. Because the extent to which this happens varies between cortical fields, the investigation of multisensory interactions can also help in understanding their relative contributions to auditory perception.

## 1 Introduction

The notion that much of sensory cortex is fundamentally multisensory in nature is now widely accepted (Ghazanfar and Schroeder, 2006). However, important questions remain as to the extent, origin, and, perhaps most importantly, *function* of inputs from other sensory areas into regions of the brain concerned primarily with modality-specific processing. Our own experience shows us that a truly unisensory experience is rare – our percept of the external world is typically formed by automatically integrating cues provided by our visual, auditory and somatosensory systems. But each of our senses contributes in different ways. While vision and audition collectively capture information about objects and events in extra-personal space, the auditory system has superior temporal resolution (Tyler and Hamer, 1990; Viemeister and Plack, 1993), whereas the visual system has greater spatial acuity (DeValois and DeValois, 1993; Brown and May, 2005). Psychophysical experiments have demonstrated that signals from different sensory modalities are often integrated in the brain in a statistically optimal fashion, with the more reliable cues having a greater influence when information is combined across the senses (Alais and Burr, 2004). This results in various illusory phenomena, such as the “ventriloquism effect”, whereby a salient visual stimulus can capture the perceived location of a sound (Howard and Templeton, 1966), and “temporal ventriloquism”, in which the number of sound bursts or the timing of a static sound can dictate the number of light flashes perceived (Shams et al., 2000) or the perceived direction of visual motion (Freeman and Driver, 2008), respectively.

Visual inputs into auditory cortex have been described in humans (e.g. Calvert et al., 1999; Giard and Peronnet, 1999; Molholm et al., 2002), non-human primates (Schroeder and Foxe, 2002; Brosch et al., 2005; Ghazanfar et al., 2005; Kayser et al., 2007), ferrets (Bizley et al., 2007; Bizley and King, 2008) and rats (Wallace et al., 2004). Because visual localization is normally more accurate than auditory localization and therefore tends to dominate spatial conflicts between the two modalities, it seems reasonable to expect that one of the functions of the visual influence on processing in the auditory cortex might be to shape and refine the relatively course spatial tuning of the neurons found there. In this paper, we review recent work from our laboratory, in which we provide electrophysiological evidence that this is indeed the case.

## 2 Visual responses in the ferret auditory cortex

We performed a series of anatomical and physiological investigations in the ferret in order to quantify the incidence, distribution and cortical origins of visual inputs to the auditory cortex. Previous studies of multisensory convergence in other species have focused primarily either on local field potential or multi-unit recordings (Schroeder and Foxe, 2002; Ghazanfar et al., 2005), which meant that the existence of multisensory convergence at the neuronal level, as opposed to a mixed population of modality-specific neurons, could be demonstrated only by the presence of interactions between the different stimuli. Our experiments sought to address the question of whether individual neurons in cortex could be driven, or have their responses to sound modulated, by concurrent visual stimulation. Because it has been suggested that visual influences on neurons in the auditory cortex of awake monkeys can arise as a result of the particular behavioral task on which the animals had previously been trained (Brosch et al., 2005), we recorded from anesthetized ferrets that had not previously been used for behavior. This also reduced the possibility that changes in eye position, which, at least in monkeys (Werner-Reiss et al., 2003; Fu et al., 2004), can alter auditory cortical responses, or effects of arousal could be misinterpreted as a response to visual stimulation.

### 2.1 Distribution of sensitivity to visual stimulation in auditory cortex

**Fig. 1:**
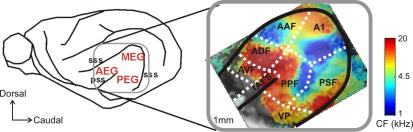
Organization of ferret auditory cortex. Auditory cortex in the ferret is located on the ectosylvian gyrus (EG), which is conventionally divided into the middle, anterior and posterior (MEG, AEG and PEG) regions. The inset shows the location of each of the auditory fields on the EG superimposed upon their sound frequency organization, as revealed using intrinsic optical imaging in a single, typical animal (from Nelken et al., 2004). A1, primary auditory cortex; AAF, anterior auditory field; PSF, posterior suprasylvian field; PPF, posterior pseudosylvian field; VP, ventral posterior field; ADF, anterior dorsal field; AVF, anterior ventral field; fAES, anterior ectosylvian sulcal field; sss, suprasylvian sulcus; pss, pseudosylvian sulcus.

As in other mammalian species, the auditory cortex in the ferret consists of multiple areas, which have been identified on the basis of anatomical studies (Bajo et al., 2007) and by the distribution of sound-evoked responses measured using intrinsic optical imaging (Nelken et al., 2004) and electrophysiological recording (Kelly et al., 1986; Bizley et al., 2005). These areas include two tonotopically-organized primary (or core) fields, the primary auditory cortex (A1) and the anterior auditory field (AAF). Three secondary (or belt) areas have been described: the posterior pseudosylvian and posterior suprasylvian fields (PPF and PSF), which are also tonotopically organized and occupy the cortex ventral to A1, plus the anterior dorsal field (ADF), ventral to AAF, which contains neurons with very broad frequency–response areas that appear to lack tonotopic order. Three other areas, the anterior ventral field (AVF), the anterior ectosylvian sulcal field (fAES), which lies adjacent to AVF within the pseudosylvian sulcus (Ramsay and Meredith, 2004; Manger et al., 2005), and the ventral posterior area (VP) are likely to be tertiary or para-belt areas. Fig. 1 shows the location of these areas in the ferret auditory cortex and illustrates their sound frequency organization, as visualized using optical imaging of intrinsic signals (Nelken et al., 2004).

In order to characterize the influence of visual stimulation on the activity of neurons in the auditory cortex, we used multi-electrode arrays to record from a total of 1024 single units in 11 ferrets, which were assigned to cortical areas A1, AAF, PSF, PPF, ADF or AVF (Bizley et al., 2007; Bizley and King, 2008). In the initial study, our stimuli comprised diffuse light flashes from a single LED positioned within the contralateral visual hemifield and broadband noise bursts delivered to the contralateral ear. The effect of presenting these very simple stimuli, either separately or together, was assessed not only by comparing the number of spikes evoked for each stimulus condition, as is typically the case in studies of multisensory processing, but also by estimating the mutual information (MI) between the responses and the stimuli that elicited them. The MI estimates allowed us to quantify how informative the response was about each stimulus condition, and also provided a more sensitive measure of the influence of the different cues (Bizley et al., 2007). This is because the MI between the stimuli and the responses was calculated in a way that exploited not just the spike counts, but also the timing of the spikes in the response, which is thought to be particularly important for the way in which auditory cortical neurons encode spatial information (Middlebrooks et al., 1998; Jenison, 2000; Nelken et al., 2005).

Using these methods, we were able to show that visual stimulation modulates the activity of neurons in all six cortical areas tested. Not surprisingly, most neurons responded to auditory stimulation with a significant change in their firing rates. Some of these also responded to the visual stimulus and, in a minority of cases, neurons were driven only by visual stimulation. In addition to these conventional visual–auditory neurons, we also encountered cases where visual stimulation, although apparently ineffective by itself, modulated the response to simultaneously-presented auditory stimulation. Such subthreshold effects have also been reported in other cortical areas (Allman et al., 2008, 2009; Meredith and Allman, 2009; Lakatos et al., 2007), and illustrate that the incidence of multisensory convergence in a given brain region is likely to be greatly underestimated if changes in spiking behavior in response to each stimulus modality presented in isolation are used as the only criterion for its presence.

**Fig. 2:**
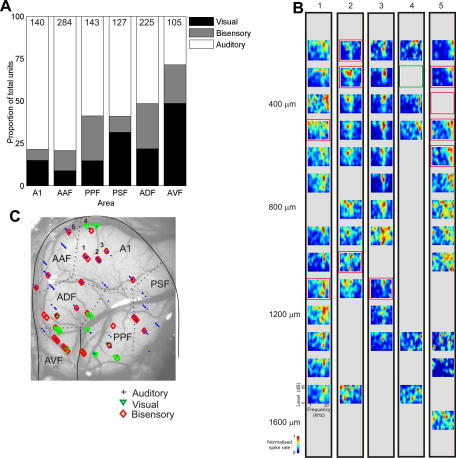
Distribution of visual sensitivity in ferret auditory cortex. (A) Bar graph showing the relative numbers of unisensory auditory (white), unisensory visual (black) and bisensory (gray) neurons recorded in each cortical field. The actual number of neurons recorded in each field is given at the top of the columns. (B) Five example penetrations (whose locations are indicated by the numbers in C). Each panel represents a single penetration made with a linear array electrode. Frequency–response areas are plotted for all sites at which units were significantly driven by pure tone stimuli. Sites in which there were significant multisensory or visual activity are outlined in red or green, respectively. (C) Distribution of response types recorded in the auditory cortex of one representative ferret. The borders between the different cortical fields are indicated by the dashed lines and were estimated from the frequency–response properties of the acoustically-responsive neurons. The locations of auditory (blue dots), visual (green triangles) and bisensory (red diamonds) neurons are plotted on the surface of auditory cortex. Note that bisensory neurons include those in which both visual and auditory stimuli produced a significant response, as well as neurons in which one modality of stimulation significantly modulated the response to the other stimulus. Based on Bizley et al. (2007) and Bizley and King (2008).

Fig. 2 shows the proportions of auditory, visual and “bisensory neurons” recorded in each of the cortical fields examined. The bisensory category included those neurons in which visual stimulation either evoked a significant change in firing rate or merely modulated the response to auditory stimulation. Even in the primary cortical fields, A1 and AAF, ∼20% of the neurons tested were sensitive to visual stimulation, and were classed as either unisensory visual or bisensory. Fig. 2A illustrates that as we ascend the cortical hierarchy, the proportion of neurons assigned to each of these two categories increases, with 40–50% of neurons recorded in the secondary areas and nearly 75% of those in AVF showing sensitivity to visual stimulation. Although the proportions of unisensory visual and bisensory neurons varies between cortical fields, overall, both are more prevalent in non-primary regions of the auditory cortex. The particularly high incidence of visual and bisensory neurons in AVF is perhaps to be expected, as visual and somatosensory inputs have previously been identified in fAES, the region lying medially within the pseudosylvian sulcus (Ramsay and Meredith, 2004).

The distribution of response types across the auditory cortex of a representative ferret is shown in Fig. 2C, with the responses recorded at different depths in five of the electrode penetrations made in this animal illustrated in Fig. 2B. In rats (Wallace et al., 2004) and cats (Meredith, 2004), multisensory neurons occur predominantly at the borders between visual, auditory and somatosensory cortex. This is obviously consistent with modality-specific afferent projections to each of these areas spilling over into an adjacent area where another sensory modality is represented. Within the different areas of the ferret auditory cortex, however, we observed no obvious pattern in the distribution of neurons sensitive to visual stimuli. Some penetrations containing neurons whose activity was influenced by visual stimuli were found toward the edge of the auditory field in question (e.g. penetrations 4 and 5 in Fig. 2B and C are close to the dorsal tip of the middle ectosylvian gyrus), whereas others were located more centrally (e.g. penetrations 1–3) and well away from sulcal regions that have previously been shown to receive non-auditory inputs. Moreover, the auditory response properties, such as their tuning for sound frequency, did not differ between auditory and bisensory neurons (Bizley et al., 2007). While this raises important questions about the integration of modality-specific auditory and visual response properties in these neurons, it does seem likely that visual inputs will, in some way, influence the contributions of the different cortical areas to auditory processing and perception.

The simple and fairly intense stimuli – contralateral noise bursts and global light flashes – used to uncover visual–auditory interactions in the ferret auditory cortex are unlikely to be optimal for many neurons. It is probable that at least some of the unisensory visual neurons would have responded to appropriate acoustic stimulation. It may also be the case that more ethologically relevant stimuli would have uncovered more extensive visual–auditory interactions. Recordings from the macaque auditory cortex, for example, have shown that responses to vocalizations can be enhanced or depressed when the animals view matching facial expressions but not when artificial visual stimuli are used instead (Ghazanfar et al., 2005). A recent study of auditory–visual interactions in Macaque auditory cortex revealed roughly the same proportion of multisensory responses when either artificial or more naturalistic stimuli were used (Kayser et al., 2008), but for individual recording sites the enhancement observed often depended on the precise choice of stimulus.

Further evidence for the widespread cortical distribution of multisensory interactions is provided by the finding that the prevalence of subthreshold auditory influences on visual responses in ferret area 21 increased substantially when local inhibition was blocked (Allman et al., 2008). It is also well known, at least for the superior colliculus, that multisensory interactions are most apparent when near threshold levels of stimulation are used (Stein et al., 1988). Level effects are probably even more important in the auditory cortex, where non-monotonic response-level functions predominate. It is therefore likely that the conditions under which our recordings were carried were not the most favorable for revealing the true extent and nature of multisensory convergence in auditory cortex. Nevertheless, these experiments allowed us to gain a rapid insight into the effects of visual stimulation on the activity of neurons in the auditory cortex, which, in many cases, became apparent only when visual and auditory cues were presented together.

### 2.2 Crossmodal interactions can be facilitatory or suppressive

In a small number of cases, we observed highly non-linear facilitatory interactions whereby a neuron might not respond at all, or only very weakly, to either form of unisensory stimulation, but responded reliably when both were presented simultaneously (an example can be seen in Bizley et al., 2007, Fig. 2F). Although response facilitation is generally much weaker, such effects can nonetheless give rise to a marked increase in the population response of the cortical area in question (Allman et al., 2009). In keeping with other studies (e.g. Dehner et al., 2004), we also recorded neurons in which one modality of stimulation had a suppressive effect on the responses evoked by the other. Moreover, by varying the relative onsets of the visual and auditory stimuli, we found a small subset in which the specific temporal relations of the stimuli unmasked interactions that were not apparent when they were presented simultaneously. This has also been observed in previous studies of both the cortex (Lakatos et al., 2007) and superior colliculus (King and Palmer, 1985; Stein et al., 1988), which have shown that manipulations of the temporal or spatial relationships between multisensory stimuli can change response enhancement to suppression and *vice versa*. While further studies will be needed in order to address the functional significance of this wide range of multisensory influences, these effects again highlight the importance of presenting the appropriate stimulus combinations in order to reveal the bisensory properties of cortical neurons.

### 2.3 Information in spike timing is crucial for revealing multisensory interactions

The MI analysis of the responses of ferret cortical neurons allowed us to assess the relative contributions of spike count and spike timing to their sensitivity to multisensory stimulation (Bizley et al., 2007). This was done by calculating the MI from two reduced spike statistics – the spike count and the mean spike latency, two measures which together have previously been shown to capture the full information available in the full spike pattern of neurons in the auditory cortex for both simple and naturalistic sounds (Nelken et al., 2005). The mean response latency is the average latency of all spikes in the response window, and equals the first spike latency when there is only one spike. This analysis revealed that, for both unisensory and bisensory auditory–visual stimulation, just over half the neurons tested transmitted more information in the timing of their responses than in their spike counts. This was particularly the case when the number of evoked spikes was low, and accords with previous studies showing that the time-locked activity in the responses of auditory cortical neurons is particularly informative about the identity (Nelken et al., 2005; Schnupp et al., 2006) and location (Middlebrooks et al., 1998; Jenison, 2000; Nelken et al., 2005) of sounds. For prolonged stimulation, the precise temporal pattern of spikes is likely to become progressively more important as it becomes increasingly less meaningful to calculate reduced statistics such as spike count or mean spike latency. In such situations the role of spike timing relative to the on-going oscillations in the local field potential may play an important role in encoding sensory information (Lakatos et al., 2007; Kayser et al., 2009).

## 3 Visual responses likely originate in visual cortex

Multisensory responses in auditory cortex could arise from converging inputs from modality-specific areas, or be inherited from either cortical or subcortical multisensory sources. Our anatomical data indicate that projections from visual cortical areas are likely to be involved. This is in line with other studies that have described the connectivity of auditory cortical areas with other sensory systems (Vaudano et al., 1991; Cappe and Barone, 2005; Budinger et al., 2006, 2007; Hackett et al., 2007; Smiley et al., 2007; Allman et al., 2008; Campi et al., 2009; Cappe et al., 2009).

Previous studies have demonstrated the existence of a direct projection from A1 to primary visual cortex (V1) in primates (Falchier et al., 2002; Rockland and Ojima, 2003). By placing deposits of highly sensitive neuronal tracer into physiologically-defined auditory cortical fields in ferrets, we found that a sparse projection exists from area V1 to both A1 and AAF (Bizley et al., 2007). This projection originates mostly from regions of V1 thought to represent the peripheral visual field (Cantone et al., 2005). Whilst the core auditory areas A1 and AAF in the carnivore brain are likely to be homologous to primate core auditory cortex, identifying homologies between belt and para-belt areas in the primate and carnivore brain is very difficult. A different pattern of innervation was found for the non-primary areas of the auditory cortex (Bizley et al., 2007). Thus, tonotopically-organized fields PPF and PSF are relatively heavily innervated by areas 20a and 20b, which are thought to be involved in processing information related to visual stimulus identity (Cantone et al., 2005). By contrast, the anterior areas located ventral to AAF (ADF, AVF and fAES) are innervated by the suprasylvian cortex (SSY). Neurons in SSY are sensitive to visual motion, and this area has been identified as a possible ferret homologue of primate MT and is therefore part of the visual “where” processing stream (Philipp et al., 2005). The posterior parietal cortex, another component of the “where” processing stream (Manger et al., 2002), also sends a small projection to the auditory fields on the anterior bank of the ectosylvian gyrus (Bizley et al., 2007). In addition, we found that the auditory belt areas are innervated by the suprageniculate nucleus, a multisensory thalamic nucleus, which is consistent with reports in other species (Budinger et al., 2006, 2007; Hackett, 2007).

**Fig. 3:**
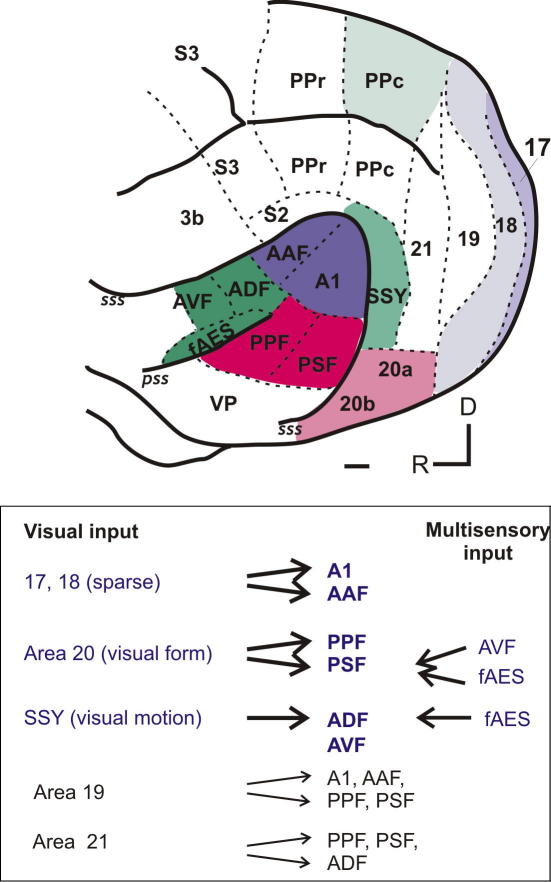
Summary of inputs from visual cortex to auditory cortex. The lateral view of the ferret brain shows the main source of visual cortical input for each region of the auditory cortex. Visual areas 17 and 18 innervate the auditory core areas (blue), visual area SSY and the PPc principally innervate the anterior belt and para-belt areas (green), while visual areas 20a and 20b project to the posterior auditory belt areas (red). A summary of the cortical multisensory and visual inputs to each auditory field is provided in the lower panel. There are also very sparse inputs from visual areas 19 and 21 to various auditory areas. PPr, rostral posterior parietal cortex; PPc, caudal posterior parietal cortex; SSY, suprasylvian cortex; 3b, primary somatosensory cortex; S2, secondary somatosensory cortex; S3, tertiary somatosensory cortex; D, dorsal; R, rostral. Other abbreviations as in Fig. 1. Scale bar is 1 mm. Based on Bizley et al. (2007).

These anatomical findings are summarized in Fig. 3. The relative strength of the connections seems to be broadly consistent with the extent to which visual stimulation can influence the activity of neurons in the different regions of the auditory cortex. Thus, the sparse inputs to A1 and AAF might be sufficient to account for the minority of neurons found there that respond to or are modulated by visual stimuli. The non-primary auditory cortical areas examined all receive more substantial inputs from higher-level visual areas and, in turn, contain a higher proportion of visually-sensitive neurons.

These data also raise intriguing predictions about the functional consequences of this differential targeting of the auditory cortex by visual inputs. In the visual cortex, spatial and non-spatial stimulus properties are, to a large degree, processed by separate functional streams (Ungerleider and Haxby, 1994). If the connections between visual and auditory areas reflect these functional differences, we might expect that the auditory cortex should exhibit a comparable parallel organization. However, a recent investigation into the representation of the pitch, timbre and azimuthal location of sounds in different areas of the ferret auditory cortex has revealed that most neurons are sensitive to at least two of these stimulus parameters (Bizley et al., 2009). Despite these overlapping distributions, some inter-areal differences were found in that study, with pitch and timbre sensitivity – properties associated with auditory object identification – being greatest in the primary and posterior cortical areas, while spatial sensitivity was most apparent in A1 and in the region around the pseudosylvian sulcus (Bizley et al., 2009). These physiological results broadly support the idea that there might be some functional specialization within ferret auditory cortex that mirrors the differences observed in visual cortical innervation.

## 4 Visual inputs can enhance spatial processing in the auditory cortex

The significance of widespread inputs from other modalities into early sensory cortices remains unclear. These inputs, which are often modulatory in nature, may improve signal processing by enhancing the response to the primary sensory modality in the presence of other cues arising from the same source (Lakatos et al., 2007; Allman et al., 2008). Visual inputs to auditory cortex are also likely to have more specific functions, such as in audiovisual communication (Ghazanfar et al., 2005; Pekkola et al., 2005). Given the pronounced influence of visual cues on auditory localization (Stein et al., 1988; King, 2009), we hypothesized that multisensory stimulation might also enhance the spatial sensitivity of neurons in auditory cortex.

We examined this electrophysiologically by recording the responses of neurons to broadband noise presented in virtual acoustic space (King et al., 2001) and to LEDs located at 15° intervals in the horizontal plane from +5°, just on the ipsilateral side of the frontal midline, to −95°, on the side contralateral to the recording site (Bizley and King, 2008). Both stimuli were presented from corresponding locations either simultaneously or in isolation. The MI was estimated between the neuronal responses and the location of the stimuli, again in a manner that allowed potential spike timing information to be exploited. In this part of the study, recordings were restricted to cortical areas A1, AAF, ADF, PPF and PSF.

As predicted on the basis of the origin of its visual cortical inputs, neurons in ADF were more sensitive for light-source location, and in fact also for sound-source location, than those in the other auditory cortical areas. We examined whether spatially and temporally coincident auditory and visual stimulation altered the amount of spatial information available from the responses relative to the most informative unisensory stimulus. Just over one half of the neurons tested across all regions of the auditory cortex showed a significant crossmodal interaction, with combined visual–auditory stimulation resulting in an increase in spatial information in two-thirds of those neurons and a decrease in the remaining one-third (Bizley and King, 2008).

**Fig. 4:**
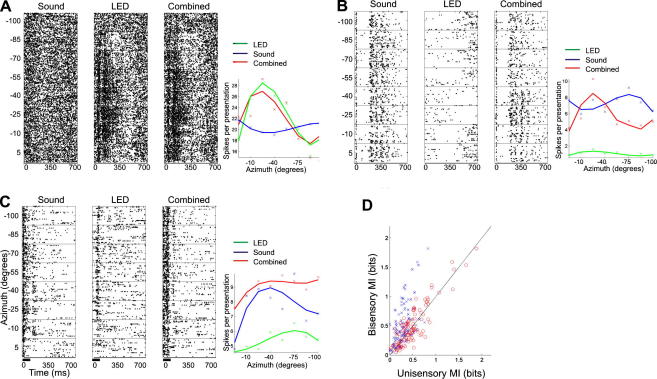
Raster plots and spatial receptive fields (based on spike counts) for three example neurons. (A) A unisensory visual neuron whose response was unaffected by simultaneous auditory stimulation. (B) A neuron whose auditory spatial receptive field was sharpened by simultaneous visual stimulation. (C) A neuron in which the bisensory response was less spatially sensitive than either unisensory response. (D) Scatter plot showing the mutual information (in bits) transmitted about the location of the stimulus for unisensory visual stimulation (red) and unisensory auditory stimulation (blue) against that obtained with bisensory stimulation. In the case of the blue crosses, most of the points lie above the *x* = *y* line, indicating that bisensory stimulation increased the spatial information in the response relative to that produced by unisensory auditory stimulation. Based on Bizley and King (2008).

Classically, bisensory integration is examined either with both stimuli at the “best” location, or with one modality of stimulation at the best location and the other systematically varied about it. These studies frequently reveal that spatially aligned bisensory stimulation can produce significant response enhancement relative to either unisensory stimulus alone. Here, however, the stimuli were always aligned. An example of a neuron in which no crossmodal interaction was observed is shown in Fig. 4A. In this case, the neuron was insensitive to auditory stimulation, as this had no effect on the activity of the neuron when presented alone or on the contralateral visual receptive field. In other neurons, however, combined visual–auditory stimulation produced a much more spatially informative response than either modality of stimulation did alone. For example, over the range of values tested, changes in sound-source azimuth did not significantly alter the firing rate of the acoustically-responsive neuron in Fig. 4B. The activity of this neuron appeared to be weakly suppressed by visual stimulation, again in a location-independent fashion. But when the two stimuli were paired, we observed a significant increase in the spatial information available in the response, which can be seen in the raster plot and the azimuth–response plot as an increased spike rate to a fairly restricted region of space in the anterior contralateral quadrant. An example of the third group of neurons, in which bisensory stimulation resulted in a decrease in transmitted spatial information is shown in Fig. 4C. In these neurons, bisensory stimulation frequently produced significant response enhancement. However, this increase in spike rate was often at the cost of having a broader receptive field relative to either unisensory condition.

Bisensory neurons that responded to each modality when presented in isolation typically exhibited broad auditory spatial sensitivity, but more restricted visual receptive fields. In these cases, the spatial MI value obtained with bisensory stimulation was usually far greater than with auditory stimulation alone, but quite similar to that estimated for the visual response. This can be seen in Fig. 4D, which plots, for each bisensory unit, the MI values obtained under either bisensory or unisensory stimulation.

**Fig. 5:**
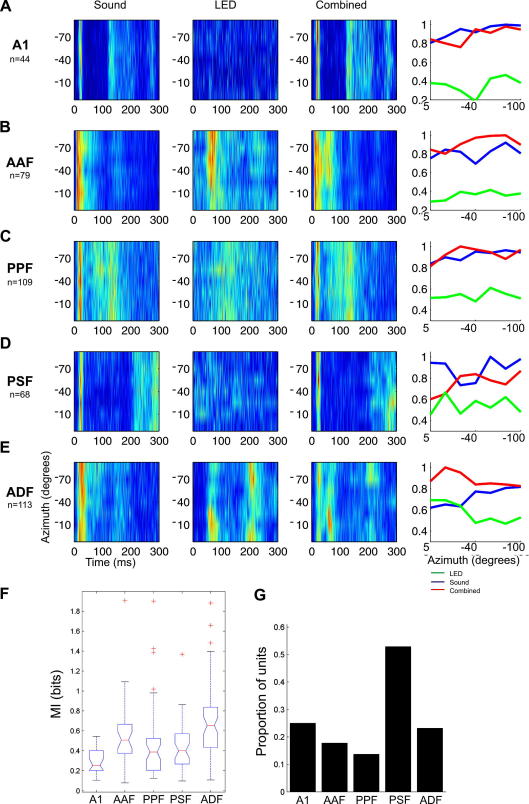
(A–E) Population azimuth–response plots (based on spike counts) for each of five auditory cortical fields (A1, AAF, PPF, PSF and ADF). The colour scale indicates the normalized spike rate plotted at different azimuthal angles as a function of time (each stimulus came on at time 0 for 100 ms). The first column shows the population response to auditory stimulation, the second to visual stimulation and the third to combined visual–auditory stimulation, while the normalized spike rates for each are combined in the azimuth–response profiles in the last column. (F) Box-plot showing the MI transmitted by neurons in each of these five cortical areas about the location of spatially and temporally coincident bisensory stimulation. The spatial MI values obtained for ADF were significantly higher than all the other cortical areas. (G) The proportion of neurons in each cortical area for which the responses to bisensory stimulation conveyed more information about stimulus location than the response to sound alone. Based on Bizley and King (2008).

Fig. 5 shows the average spatial receptive field for auditory, visual or bisensory stimulation based on the responses of all of the neurons recorded in each cortical area. While constructing population receptive fields in this fashion will obviously poorly reflect individual spatial tuning if different neurons are tuned to different locations, this approach is justified because neurons in the auditory cortex are most commonly tuned to contralateral sound locations and has been used before for displaying the auditory population response in different species (Mrsic-Flogel et al., 2003; Woods et al., 2006; Harrington et al., 2008). The auditory responses show little modulation with sound-source location, with only 23% of neurons transmitting significant spatial information. This reflects the relatively high sound levels used and the limited range of azimuthal locations tested. Indeed, we found that more than half the neurons transmitted significant information about sound-source location when the range of virtual sound directions tested was increased to just beyond the full frontal hemifield (Bizley and King, 2008). Nevertheless, Fig. 5 clearly illustrates the fact that acoustically-responsive neurons in AAF (Fig. 5B) and particularly in ADF (Fig. 5E), which exhibit the sharpest visual spatial tuning among the auditory cortical areas, show enhanced spatial sensitivity in the presence of spatially-congruent bisensory stimuli.

Fig. 5F shows the amount of spatial information available in the responses recorded in each of the five cortical areas to combined visual–auditory stimulation, again highlighting how neurons in ADF have greater spatial sensitivity than those in the other areas. We examined the proportion of neurons in each cortical field whose responses transmitted more spatial information when a visual stimulus was added to the auditory stimulus, compared to that estimated from the response to the sound alone. Fig. 5G plots these data and illustrates that between 10% and 20% of neurons in each cortical field transmit more information about stimulus location in the presence of bisensory stimuli, with the majority of others conveying about the same number of bits of information in the unisensory and bisensory conditions. PSF is the exception to this where over 50% of neurons show enhanced spatial coding under these circumstances, although the overall MI values are much lower than those in ADF.

The finding that combined visual–auditory stimulation enhances spatial tuning in a sizable subpopulation of neurons strongly suggests that one function of visual inputs to auditory cortex is to facilitate the localization of auditory stimuli. By the same token, it seems likely that such activity might provide the basis for crossmodal spatial illusions such as ventriloquism, which is thought to originate from the auditory cortex (Recanzone, 1998). Of the five cortical areas examined, we found that neurons in ADF transmit the most spatial information for auditory, visual and bisensory stimuli (Fig. 5; Bizley and King, 2008). This fits well with the anatomical data, in that ADF is innervated by neurons in field SSY, a visual “where” processing area (Philipp et al., 2005), supporting the idea that inputs from visual cortex contribute to the visual activity recorded in auditory cortex. Although PSF receives inputs from visual areas 20a and 20b, which are more concerned with non-spatial visual processing (Manger et al., 2004), rather than from SSY, this posterior belt area contained the largest number of neurons that showed an increase in spatial information when the responses to auditory and bisensory stimulation were compared. This may seem a puzzling finding, but PSF is reciprocally connected with fAES (J.K. Bizley, F.R. Nodal, V.M. Bajo and A.J. King, unpublished observation), providing a potential link between areas that appear to be involved primarily in stimulus localization or identification. Integration of both spatial and non-spatial cues is critical for grouping together sounds that originate from a particular source and for segregating sounds that originate from different sources. Thus, by enhancing the spatial sensitivity of neurons in areas that may be less well specialized for sound localization *per se*, visual inputs could provide an important role in the representation of auditory objects.

## 5 Concluding remarks

The physiological and anatomical studies reviewed here have shown that many neurons in the ferret auditory cortex have multisensory properties and integrate auditory and visual signals in ways that change their spatial tuning characteristics. We have focussed solely on visual influences, because of the role shared by these two systems in representing objects and events in the surrounding environment, but it seems likely that somatosensory and perhaps other modality inputs also contribute to signal processing in the auditory cortex. Although we cannot rule out the possibility that the visual sensitivity of these neurons may, at least in part, have a subcortical origin, we believe the most likely source of these inputs is the visual cortex. Indeed, the extent to which visual stimuli can either drive auditory cortical neurons in different auditory fields, or have a subthreshold modulatory effect on their responses to sound, seems to correlate well with the size of the projections from particular visual cortical areas. To conclusively demonstrate this, however, would require an investigation of the effects of inactivation of individual visual cortical areas.

It is well established in ferrets, as well as other carnivores and primates, that an intact auditory cortex is necessary for normal sound localization (Kavanagh and Kelly, 1987; Malhotra et al., 2004; Smith et al., 2004; Malhotra and Lomber, 2007). Our finding that spatially-congruent visual cues can enhance spatial processing throughout the auditory cortex suggests that these interactions may well underlie the pronounced influence of vision on the perception of auditory space, although further experiments will be required to test this directly. Interestingly, the amount of information conveyed about source location varies between the auditory fields and according to the functions of the areas of extrastriate visual cortex to which they are connected. Characterizing those projections, and the way in which they alter the response properties of neurons in the auditory cortex, is therefore likely to provide valuable insights into the still poorly understood functions of the different areas that constitute the auditory cortex.
